# Inflammatory Blood Biomarkers Are Associated with Long-Term Clinical Disease Severity in Parkinson’s Disease

**DOI:** 10.3390/ijms241914915

**Published:** 2023-10-05

**Authors:** Dagmar H. Hepp, Thecla A. van Wageningen, Kirsten L. Kuiper, Karin D. van Dijk, Linda P. Oosterveld, Henk W. Berendse, Wilma D. J. van de Berg

**Affiliations:** 1 Department of Anatomy and Neurosciences, Amsterdam UMC Location Vrije Universiteit Amsterdam, de Boelelaan 1108, 1081 HZ Amsterdam, The Netherlands; d.hepp@amsterdamumc.nl (D.H.H.);; 2 Department of Neurology, Amsterdam UMC Location Vrije Universiteit Amsterdam, de Boelelaan 1117, 1081 HZ Amsterdam, The Netherlands; h.berendse@amsterdamumc.nl; 3 Amsterdam Neuroscience, Program Neurodegeneration, Amsterdam UMC Location Vrije Universiteit Amsterdam, 1081 HZ Amsterdam, The Netherlands; 4 Sleep Wake Centre, Stichting Epilepsie Instellingen Nederland (SEIN), 2103 SW Heemstede, The Netherlands

**Keywords:** immune response, blood biomarkers, Parkinson’s disease, disease severity, CCL23, TGF-alpha, TNFRSF9

## Abstract

An altered immune response has been identified as a pathophysiological factor in Parkinson’s disease (PD). We aimed to identify blood immunity-associated proteins that discriminate PD from controls and that are associated with long-term disease severity in PD patients. Immune response-derived proteins in blood plasma were measured using Proximity Extension Technology by OLINK in a cohort of PD patients (N = 66) and age-matched healthy controls (N = 52). In a selection of 30 PD patients, we evaluated changes in protein levels 7–10 years after the baseline and assessed correlations with motor and cognitive assessments. Data from the Parkinson’s Disease Biomarkers Program (PDBP) cohort and the Parkinson’s Progression Markers Initiative (PPMI) cohort were used for independent validation. PD patients showed an altered immune response compared to controls based on a panel of four proteins (IL-12B, OPG, CXCL11, and CSF-1). The expression levels of five inflammation-associated proteins (CCL23, CCL25, TNFRSF9, TGF-alpha, and VEGFA) increased over time in PD and were partially associated with more severe motor and cognitive symptoms at follow-up. Increased CCL23 levels were associated with cognitive decline and the *APOE4* genotype. Our findings provide further evidence for an altered immune response in PD that is associated with disease severity in PD over a long period of time.

## 1. Introduction

There is increasing evidence for the involvement of neuroinflammation in Parkinson’s disease (PD) [1,2]. In 1988, a post-mortem study described the presence of activated microglial cells and infiltrating T lymphocytes in the substantia nigra of PD patients [3]. More recently, an association between cytotoxic T cell density and nigral dopaminergic cell loss was reported in post-mortem human brain tissue from PD patients [4]. Evidence for the direct involvement of the adaptive immune system in PD has also been documented, whereby T cells in PD were shown to react specifically to alpha-synuclein peptides [5].

Upon activation, all immune cells secrete cytokines and chemokines that can be quantified in blood plasma. Previous studies have shown that derivatives of the activated immune system measured in the blood of PD patients may serve as a non-invasive peripheral resource to reflect immune-mediated pathological changes in the central nervous system [6,7,8,9]. Recently, plasma cytokine levels were investigated in PD, PD with dementia (PDD), and dementia with Lewy bodies (DLB). A more pronounced immune response was observed in cases with dementia (i.e., PDD and DLB), compared to PD without dementia [10]. Moreover, in PD patients with a mutation in the *LRRK2* gene, a higher level of inflammatory proteins in blood was associated with a clinical subtype characterized by a more severe and broad spectrum of motor and non-motor symptoms [11]. Taking these results together, it could be hypothesized that enhanced inflammation in PD is associated with a more severe disease course and a higher risk of dementia.

To date, only a few studies have shown longitudinal associations between serum cytokine levels in PD patients and clinical function over time [12,13]. Elevated levels of pro-inflammatory associated cytokines were associated with lower cognitive scores at the baseline and a higher rate of motor progression, whereas elevated levels of anti-inflammatory cytokines predicted better cognitive performance at the baseline and stable motor function at 36 months of follow-up [12]. A limitation of this as well as other previous studies investigating inflammation in PD, is that only a limited selection of proteins was examined and that the follow-up period was relatively short [2]. More recent studies have investigated a very broad panel of plasma inflammatory biomarkers over a longer follow-up period and have identified some promising plasma biomarkers of neuroinflammation in PD that warrant further investigation [13,14,15]. The aim of the present study is to compare the expression of a broad panel of proteins involved in the immune system between PD patients and controls and to investigate the relationship between expression levels of immune-related proteins and long-term disease severity in PD. The Parkinson’s disease Biomarkers Program (PDBP) cohort and the Parkinson’s Progression Markers initiative (PPMI) cohort biomarker datasets were used for independent validation [16].

## 2. Results

### 2.1. Demographics and Clinical Data

Age was comparable between PD patients and controls, whereas gender distribution was significantly different, with more males in the PD group (*p* < 0.001; Table 1). Cognitive performance, as measured with the MMSE, was lower in PD patients, compared to controls (*p* = 0.02). The clinical data of the PD patients for whom longitudinal data were available are shown in Table 2. As expected, motor symptoms (UPDRS-III and H and Y stage; *p* = 0.01 and *p* < 0.001, respectively) were significantly more severe at follow-up measurements (T1), compared to the baseline (T0). When comparing MMSE scores, no significant cognitive decline was measured at T1 compared to T0. However, when using more sensitive neuropsychological assessments, cognitive decline was evident over time. At the baseline, all but 1 demented PD patient were cognitively unimpaired, whereas at follow-up, 5 were classified as MCI and 9 as PDD (*p* < 0.001). The AMP-PD validation cohort included 105 PD patients and 83 controls whose demographics are shown in Supplementary File S1 (Table S1).

### 2.2. Cross-Sectional Analysis: A Panel of Four Inflammatory Proteins Can Discriminate PD from Controls

**Figure 1:**
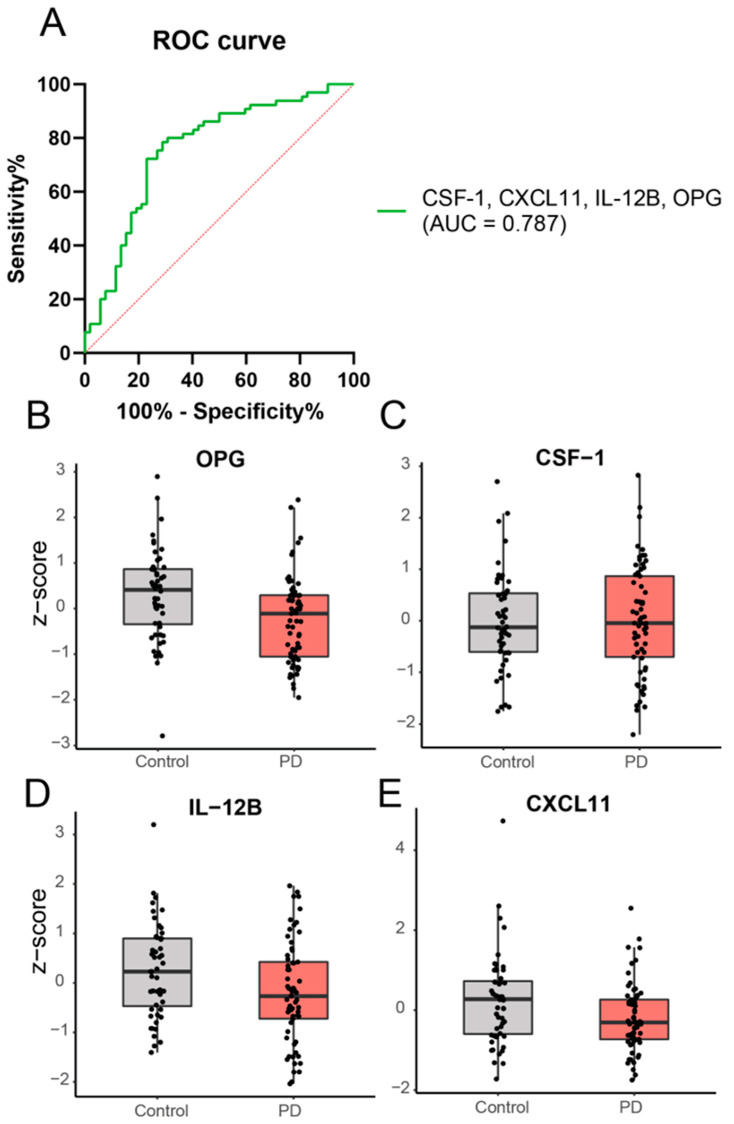
Expression levels of a combination of inflammatory proteins are significantly different in PD patients, compared to healthy controls based on binary logistic regression analysis. A subset of 4 different proteins, i.e., OPG (exp(B) = 0.09; Wald = 12.39), CSF-1 (exp(B) = 41.34; Wald = 6.79), IL-12B (exp(B) = 0.44; Wald = 5.24), and CXCL11 (exp(B) = 0.50; Wald = 6.38), could discriminate PD patients from controls. Panel (**A**) shows the ROC curve and panel (**B**–**E**) shows the expression levels of the individual proteins in PD and controls.

Three samples at the baseline (one PD and two healthy controls) were excluded from further analysis; one was excluded due to technical errors and two were because they were classified as significant outliers (Supplementary Figure S1). Of the 92 markers, 20 were excluded due to a low detection rate, resulting in successful measurement of 72 markers. PCA analysis revealed no clear clustering of PD patients relative to controls upon visual inspection. Based on the ANOVA, no individual marker was significantly different between PD and the controls. However, the binary logistic regression showed that a subset of four different proteins, i.e., IL-12B, OPG, CXCL11, and CSF-1, could discriminate PD from controls (*p* < 0.001), with an area under the curve of 0.787 (Figure 1).

### 2.3. Longitudinal Analysis: Five Inflammatory Proteins Increase over Time and Clinical Correlations

**Figure 2:**
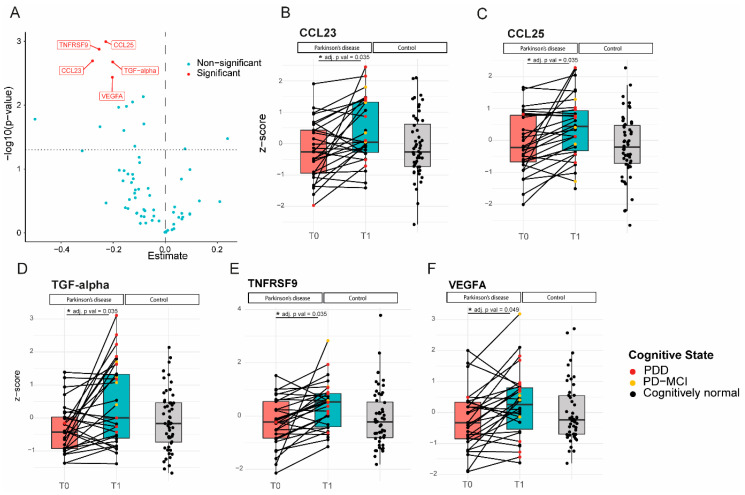
Levels of inflammatory proteins increased significantly in Parkinson’s patients over 7–10 years of follow-up. (**A**): Volcano plot; *p*-value versus the magnitude of change; (**B**–**F**): Expression profiles of all 5 proteins that increased over time for each individual subject at the two different time points, relative to the expression levels of the control subjects. CCL23, TGF-alpha, and TNFRSF9 levels (**C**,**D**,**F**) were higher in cognitively impaired subjects (PD-MCI and PDD) compared to cognitively normal PD patients. * = *p* value < 0.05.

**Figure 3:**
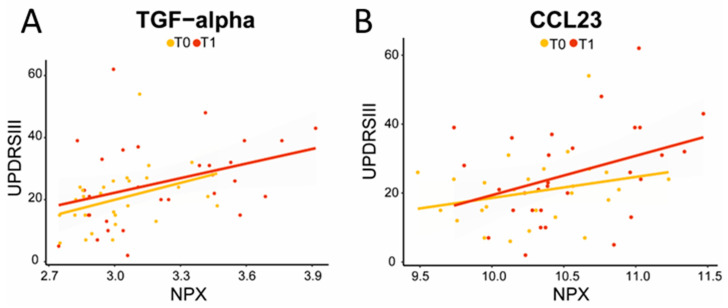
Increased TGF-alpha (**A**) and CCL23 (**B**) levels correlated with higher UPDRS III scores, indicating decreased motor performance at follow-up (TGF-alpha: ρ = 0.4, *p* = 0.02; CCL23: ρ = 0.4, *p* = 0.03). The yellow line represents T0; the red line represents T1; correlation analysis was performed on T1.

**Figure 4:**
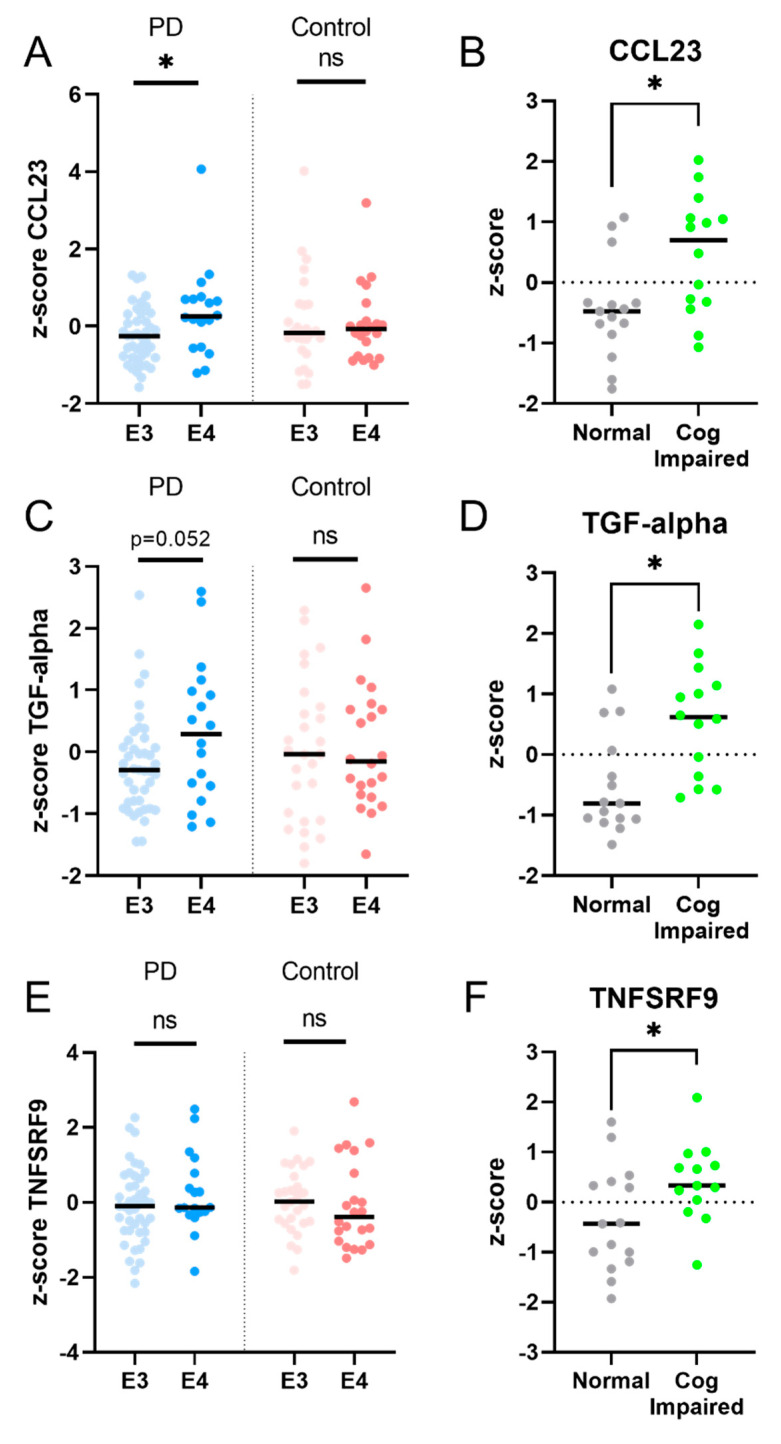
PD patients, but not controls, with the *APOE4* genotype showed increased levels of plasma CCL23 (**A**) and a trend for an increased level of TGF-alpha (**C**), but not of TNFSRF9 (**E**). PD patients who were cognitively impaired at follow-up had higher levels of CCL23, TGF-alpha, and TNFRSF9, compared to PD patients who remained cognitively unaffected (panel **B**,**D**,**F**). Legend: E3 = apolipoprotein E3 genotype; E4 = apolipoprotein E4 genotype. * = *p* value < 0.05; ns = non-significant.

The expression levels of five proteins were significantly increased at follow-up compared to the baseline in PD patients (Figure 2). Increased levels of CCL23 and TGF-alpha correlated significantly with higher UPDRS III scores at follow-up, indicating worse motor performance (CCL23, ρ (rho) = 0.4, *p* = 0.04; TGF-alpha, ρ (rho) = 0.4, *p* = 0.02; Figure 3). TGF-alpha levels correlated with higher HY disease stage at follow-up (ρ (rho) = 0.4, *p* = 0.03). Higher levels of CCL23, TGF-alpha, and TNFRSF9 were related to cognitive status at follow-up, being higher in cognitively impaired PD patients (CCL23: t(27) = 2.737, *p* = 0.01; TGF-alpha: t(27) = 3.426, *p* < 0.01; TNFRSF9: t(27) = 2.432, *p* = 0.02; Figure 2 and Figure 4). Patients with the *APOE4* genotype showed increased plasma levels of CCL23 only (t(58) = 2.508, *p* < 0.05, Figure 4). This *APOE4* genotype effect was not observed in controls.

### 2.4. Independent Validation in AMP-PD Program

We could not validate changes in any of the protein levels of our discriminatory panel with the cross-sectional analysis (corrected for age and multiple comparisons) on the OLINK neuroexplore data of AMP-PD. However, we were able to validate an increase in the protein levels of two of the five proteins, i.e., TGF-alpha and CCL25, in PD patients over time (Supplementary Figure S2). A third protein, VEGFA, also increased but only at a trend level in the independent validation cohort. CCL25 was also increased over time in control subjects over a 48-month follow-up period. Increased levels of TGF-alpha did not correlate with more severe motor symptoms at follow-up. Details of the analysis in the independent validation cohort can be found in Supplementary File S1.

## 3. Discussion

In the present study, we provide further evidence of an altered immune response in PD compared to healthy controls, measurable in blood plasma and allowing discrimination between groups. In addition, we demonstrate increasing levels of 5 out of 92 inflammatory proteins during long-term follow-up (7–10 years) in PD patients, 3 of which are associated with worse cognitive and/or motor performance. These results provide further insight into the relationship between the immune response and long-term clinical disease severity in PD.

A panel of four proteins (IL-12B, CSF-1, CXCL11, and OPG) was able to discriminate between PD and the controls. All of these proteins are associated with a pro-inflammatory environment and are involved in either the recruitment or maintenance of activated T lymphocytes [17,18,19,20,21]. The proteins osteoprotegerin (OPG), interleukin 12-b (IL-12B), and C-X-C motif Chemokine ligand 11 (CXCL11), which are all three markers associated either directly or indirectly with the T-cell response, were decreased in PD compared to controls [17]. Although reduced OPG levels have previously been reported in PD compared to controls [22], the results have been inconsistent [20]. Possibly, the adaptive immune response mediated by T cells is more elevated at the time of PD diagnosis or even prior to diagnosis. In line with this hypothesis, a longitudinal case study showed that increased T-cell mediated intracellular cytokine levels in peripheral blood mononuclear cells (PBMC) peaked around the time of PD motor diagnosis and declined thereafter [23]. This has also been shown in the central nervous system itself, as CD8^+^ T-cell infiltration was observed prior to the presence of alpha-synuclein aggregates in the substantia nigra of brain donors with incidental Lewy Body Disease (iLBD), considered to be the premotor phase of PD [4]. In addition, it has been shown that there is a selective decrease in CD4^+^ memory T-cells in PD patients which correlates with increased disease severity [24]. Indeed, lower levels of IL-12B, OPG, or CXCL11 could possibly be indicative of less activity of CD4^+^ (memory) T-cells [25,26,27]. Taken together, our results may implicate a failure of the alpha-synuclein induced adaptive immune response in PD patients, possibly allowing a-synuclein to spread further in the brain and body [28]. In contrast, the mean level of CSF-1, which is known to promote microglial proliferation and the production of pro-inflammatory mediators, was slightly higher (ns), consistent with the previous literature [17]. This could possibly indicate a difference in the innate macrophage (as reflected by CSF-1) response and adaptive T cell response (as reflected by IL-12B, OPG, and CXCL11). Future studies including patients in a prodromal phase of the disease should be performed to verify possible changes in the expression levels of markers implying a role for the adaptive immune system such as OPG, IL-12B, and CXCL11.

In our longitudinal analysis, five inflammation-associated proteins (CCL23, CCL25, TNFRSF9, TGF-alpha, and VEGFA) were increased at follow-up in PD patients. These inflammatory proteins are more related to an innate immune response rather than an adaptive immune response. CCL23 (CC chemokine ligand 23) is released by macrophages and induces the trafficking of immune cells which results in the release of pro-inflammatory cytokines [29]. Increased CCL23 levels in PD patients were associated with worse cognitive performance and more severe motor impairment. Interestingly, both in acquired brain lesions such as ischemic stroke as well as progressive neurodegenerative disorders such as Alzheimer’s disease (AD), a recent study in PD showed that CCL23 levels were elevated compared to healthy controls and it predicted a steeper cognitive decline in AD patients [13,30]. CCL23 may thus be an interesting predictive biomarker for faster disease progression in PD.

Since it has recently been demonstrated that the presence of one or more *APOE4* alleles in PD is associated with higher odds of developing dementia, as well as a shorter time to develop dementia [31], we investigated the relationship between CCL23 levels and *APOE4* genotype.

Indeed, CCL23 levels were higher in PD patients that carried an *APOE4* allele, compared to non-carriers, while this was not seen in controls. In AD, both CCL23 levels in the CSF as well as *APOE4* status are known as independent predictors for conversion from mild cognitive impairment (MCI) to dementia [30]. Also, CCL23 blood plasma levels were higher in AD patients who carried an *APOE4* allele [30]. Since the protein product of the *APOE* gene, apolipoprotein E, modulates inflammatory and immune responses in an isoform-dependent manner [32], it may be hypothesized that the *APOE4* genotype enhances the immune response as measured with CCL23 levels in both AD and PD and accelerates disease progression. In the independent validation cohort, CCL23 levels did not change over time. This could be due to the shorter follow-up period in the available AMP-PD cohort (2 years) compared to the present study (7–10 years), as well as possible differences in genotype distribution.

The levels of both CCL25 (C-C Motif Chemokine Ligand 25), which exhibits chemotactic activity towards inflammatory cells, and of tumor necrosis factor receptor superfamily member 9 (TNFRSF9; a protein that promotes T cell apoptosis), were significantly increased in PD patients at follow-up, consistent with a previous report for CCL25 [13]. The elevation of TNFRSF9 was related to worse cognitive performance at follow-up. In multiple sclerosis, TNFRSF9 appears to be involved in cell death via the activation of microglia [33,34]. Alterations in the *TNFRSF9* gene have just recently been described in relation to PD, with the *TNFRSF9* genotype being a possible disease modifier in patients with a known *DJ-1* mutation [35].

Transforming Growth Factor alpha (TGF-alpha) functions as a ligand for the epidermal growth factor receptor (EGFR), resulting in increased cell proliferation. It is an important player in the innate immune system and is expressed by astrocytes in the brain parenchyma [36]. We report an increase in TGF-alpha at follow-up, compared to the baseline, which correlated with more severe motor symptoms and cognitive performance. This is in line with previous findings in CSF [37], which is interesting since for TGF-alpha, blood and CSF levels are known to be related [38]. The correlation between TGF-alpha levels and motor symptoms at follow-up was not present in the validation cohort. This could be due to clinical differences, e.g., motor performance was worse in the validation cohort and/or differences in the follow-up period.

Another protein involved in the (brain) innate immune system, Vascular Endothelial Growth Factor A (VEGFA), also showed increased levels in the blood of PD patients over time. Interestingly, in a very recent animal study, increased production of VEGFA was reported in astrocytes that were treated with oligomeric alpha-synuclein [39]. Upregulation of VEGFA has also been reported in reactive astrocytes in the substantia nigra of PD patients [40]. The increased production and release of VEGFA may contribute to the degradation of the blood–brain barrier (BBB), making the brain even more vulnerable to oligomeric alpha-synuclein. Clinically, higher VEGFA levels have previously been associated with the development of dementia in an elderly population [41]. However, in the current study, no association was observed between the increase in VEGFA levels and changes in any of the clinical measures. This may be due to our relatively small sample size in the longitudinal analysis and a shorter follow-up period compared to the above study, i.e., 7–10 years compared to 17 years [41]. To our knowledge, a direct relationship between alpha-synuclein and the other markers that were associated with disease severity in our cohort, i.e., CCL23, CCL25, TNFRSF9, and TGF-alpha, has not been investigated to date. This would be an interesting topic for future research.

Taken together, our data seem to imply that a failure of the adaptive immune system may occur in PD compared to controls, whereas PD disease progression and especially cognitive decline are associated with an enhanced innate immune response characterized by macrophage or local glial activation, similar to what is observed in other neurodegenerative diseases.

Several limitations should be considered in the interpretation of the present results. First, our study had a small sample size. However, we were able to validate the increase over time of two out of the five proteins in an independent cohort. Second, we had no follow-up measurements in controls. This is particularly relevant since age is known to significantly influence cytokine expression levels, such as CCL25 and VEGFA [42]. However, we did have follow-up measurements of controls in the validation cohort and only CCL25 levels increased over time in controls as well and may thus not be PD-specific. Third, gender could not be included in the binary logistic regression due to significant gender differences between groups. This might have affected the outcome as the immune system is known to display sex-specific differences [43]. Indeed, OPG levels were significantly lower in men with PD compared to women with PD in the present study cohort, which may have influenced our results. However, the other three inflammatory proteins did not differ in levels between the sexes. Lastly, differences in immune cell populations were not measured and/or defined in the present study and some promising plasma inflammation biomarkers from previous studies, such as IL6 [13], were not included in the OLINK panel. However, a positive point of our study is that we managed to find meaningful differences possibly reflecting pathological processes in protein levels in blood plasma which is more easily acquired than CSF.

## 4. Conclusions and Future Directions

In conclusion, our data-driven and exploratory study provides further evidence for an altered immune response in PD that can be measured in blood plasma. A panel of four proteins particularly involved in the adaptive immune response, i.e., IL-12B, CSF-1, CXCL11, and OPG, was able to discriminate between PD and controls. Five proteins involved in the innate immune response increased in PD patients during long-term follow-up. Three of these, namely CCL23, TGF-alpha, and TNFRSF9, were related to disease severity, making them promising immune-related biomarkers for disease progression in PD.

Future mechanistic studies investigating a direct link between these peripheral immune markers and alpha-synuclein are warranted. One possible approach would be to incubate peripheral mononuclear blood cells from clinical cohorts with alpha-synuclein and measure the immune markers. Ideally, such a study would include patients at different stages of PD, including prodromal cases, to follow changes in the immune system throughout the course of the disease. In this way, the hypothesis that neuroinflammation in PD begins with a failed adaptive immune response and then involves an ongoing low-grade innate immune response could be tested. Ultimately, a better understanding of neuroinflammation in PD is of the utmost importance to advance the development of novel immunomodulatory approaches in PD treatment [44].

## 5. Materials and Methods

### 5.1. Study Population and Clinical Assessment

PD patients and age-matched healthy controls participated in an Amsterdam UMC cohort study of which recruitment and inclusion has been described elsewhere [45]. Briefly, PD patients were recruited from the outpatient clinic for movement disorders at the Amsterdam UMC, the Netherlands. The healthy controls were recruited through an advertisement of the Dutch Parkinson Foundation. PD patients fulfilled the clinical diagnostic criteria of the UK PD Brain Bank [46]. Patients were included if no signs of moderate dementia were detectable upon Mini-Mental State Examination (MMSE score < 19) at the baseline. After 7–10 years of follow-up, patients were included for repeated clinical assessment and blood sampling. Exclusion criteria were a change in PD diagnosis, inability to understand the study procedures, and/or having an MMSE score of <19. The local ethics committee approved the study and written informed consent for the use of clinical data and biomaterial for research purposes was obtained from all participants.

**Figure 5:**
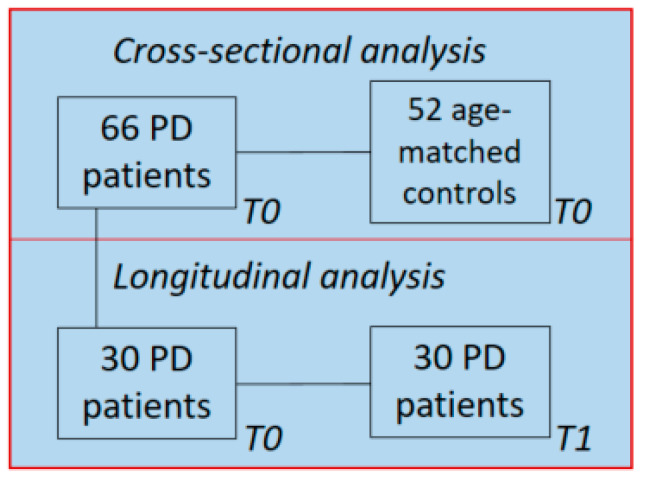
Study population: 30 PD patients’ follow-up data and blood samples were available 7–10 years after the baseline and were used for longitudinal analysis.

The disease duration was calculated based on a subjective estimate of the time since the first occurrence of the characteristic PD motor symptoms. The Unified Parkinson’s Disease Rating Scale part III (UPDRS-III) and the Hoehn and Yahr (HY) classification were used to quantify the severity of parkinsonism and disease stage, respectively, and were assessed in the “on” state. The education level was determined using the Verhage score which ranges from level 1 (elementary school not finished) to 7 (university). Cognitive status was examined using the MMSE at the baseline (T0). At follow-up (T1), an extensive neuropsychological examination was performed and resulted in a diagnosis of ‘normal cognitive status’, ‘PD with mild cognitive impairment’ (PD-MCI), or ‘PD with dementia’ (PDD). PD-MCI was diagnosed if the performance on at least two neuropsychological tests in at least one cognitive domain was impaired (i.e., >1.5 SD below normative means) [47,48]. PDD was diagnosed if test scores were >2 SD below normative means in ≥2 domains. In addition, the Levodopa Equivalent Daily Dose (LEDD) was calculated for each patient as described elsewhere [49]. For the present study, we only included the data of PD patients of whom blood-derived EDTA plasma samples as well as extensive neuropsychological data at follow-up were available (Figure 5).

### 5.2. Validation in PDBP and PPMI Cohorts

For validation purposes, we used proteomics data of the Accelerating Medicines Partnership Parkinson’s Disease (AMP-PD) initiative, which consists of data from plasma samples of the Parkinson’s Disease Biomarkers Program (PDBP) cohort and Parkinson’s Progression Markers Initiative (PPMI) cohort. The data are available at https://amp-pd.org/data/targeted-proteomics-data (accessed on 1 July 2023), as summarized in Supplementary File S1. Data was accessed on 1 July 2023.

#### Plasma samples and Proximity Extension Technology (PEA) by OLINK

EDTA blood plasma sampling was performed as described earlier [45]. EDTA plasma samples were centrifuged at 1800× *g* at 4 °C for 10 min and were aliquoted and stored in −80 °C within 2 h. To quantify the levels of inflammation related proteins in plasma, the 96 Target Inflammation Panel of OLINK (Uppsala, Sweden) was used. This panel consists of 92 markers for proteins that are either directly or indirectly involved in inflammatory processes. The 96 Target Inflammation panel was chosen based on the inclusion of proteins specifically associated with either innate or adaptive immunity within the panel. The list of these proteins is available at the company’s website; https://www.olink.com/products/target/inflammation/ (accessed on 1 July 2023). Nunc™ 96-Well Polypropylene Storage Microplates in combination with MicroAmp™ Optical Adhesive Film (Applied Biosystems, Waltham, Massachusetts, US, catalog number #4311971 was used for sample transport. Blood plasma samples stored at −80 °C were thawed on ice and 40 µL for each sample was plated following a randomized and centered plate-design. Plate controls, negative controls, and sample controls were provided by OLINK. Plate controls included samples of healthy blood donors and were used as references for inter-plate differences. The negative control consisted solely of buffer to set the background noise and determine the limit of detection (LOD). Sample controls consisted of external plasma samples for the inter- and intra-plate precision of each assay.

For the expression level measurements, a Proximity Extension Technology (PEA) was used, after which real-time qPCR allowed for simultaneous read-out of the 92 markers per sample. Normalized Protein Expression (NPX) is OLINK’s arbitrary unit in the Log2 scale used to present normalized protein levels per marker per sample. Both inter-plate and intra-plate controls were included for quality control and data validation of the analysis. NPX values were filtered and selected based on exceeding the determined LOD threshold. Moreover, protein markers were only included for quantitative analysis if less than 10% of their within-sample NPX values were below the LOD. Distribution plots and Principal Component Analysis was used to evaluate unsuccessful measurements and to identify outliers that were not included in the analysis.

Independent validation of inflammatory protein biomarkers was performed using data from the AMP-PD program which exploited the OLINK Explore 1536 panel (including the 384 Cardiometabolic, 384 Inflammation, 382 Neurology and 384 Oncology panel; https://olink.com/products-services/explore/ (accessed on 1 July 2023)). In the explore panels, next-generation sequencing was used as a read-out instead of real-time qPCR [50]. All of the proteins in the 96Target Inflammation panel are part of the OLINK Explore 1536 panel.

### 5.3. APOE Genotyping

Since the *APOE4* genotype is associated with an increased risk of cognitive decline in PD [51], we evaluated the relationship between protein plasma values of proteins that were related to cognitive decline at follow-up and the *APOE4* genotype. DNA samples were genotyped using the neurochip [52]. *APOE* genotypes were determined based on combinations of the absence or presence of the SNPs rs429358 and rs7412. Data were analyzed using Plink 2.0.

### 5.4. Statistical Analysis

All statistical analyses were performed with R studio (version 4.0.3) or IBM SPSS Statistics (version 26). A *p*-value below 0.05 was considered significant. Descriptive variables were compared between groups using the Pearson’s Chi squared test for categorical data, a two-sided T-test for normally distributed continuous data, or a Mann–Whitney U test for non-normally distributed continuous data when appropriate.

Principal Component Analysis (PCA) was performed to visualize clustering of protein panels between groups. A two-sided ANOVA was performed to detect individual protein differences between PD patients and controls adjusted for age. To correct for multiple testing, the Benjamin-Hochberg False Discovery Rate (FDR) calculation (q < 0.05) was used. In addition, a binary logistic regression analysis with forward conditional selection was carried out in SPSS with inflammatory markers as predictors and age as a covariate. We tested for multicollinearity by determining the variance inflation factor (VIF) of all proteins, whereby a VIF factor of 2.5 or greater would indicate considerable multicollinearity [53]. Thereafter, a receiver operating characteristic (ROC) curve analysis was carried out resulting from the predictive values from the binary logistic regression model to detect the area under the curve.

For the longitudinal analysis, differences in group characteristics were measured in the same way as described above, only a paired sampling design was used. Therefore, a paired *t*-test was used to calculate differences between participants over time. Correlation analysis between protein expression levels and clinical evaluations was carried out using Spearman’s Correlation coefficient (UPDRS III and HY stage) and a two-sided T-test (cognitively intact vs. cognitively impaired).

## Figures and Tables

**Table 1:** Demographics and clinical measures of PD patients and controls at the baseline.

	PD Patients	Healthy Controls
N	66	52
Male nr. (%)	44 (67%) *	18 (35%)
Age (years)	62 ± 10	63 ± 7
Verhage education score (1/2/3/4/5/6/7)	0/1/1/7/14/7/10	n/a
Disease duration (years)	6 ± 5	n/a
H and Y stage (1/1.5/2/2.5/3/4/5)	5/5/32/17/7/0/0	n/a
UPDRS-III score	22 ± 8	n/a
MMSE score (range)	28 ± 2 (23–30) *	29 ± 1 (25–30)
LEDD	417 ± 453	n/a

Data are presented as mean ± standard deviation unless indicated otherwise. Abbreviations: H and Y stage = Hoehn and Yahr stage, UPDRS-III = Unified Parkinson’s Disease Rating Scale III, MMSE = Mini-Mental State Examination, LEDD = Levodopa Equivalent Daily Dose, n/a = not applicable. * Difference is statistically significant (*p* < 0.05).

**Table 2:** Demographics and clinical measures of PD patients of which follow-up data were available (N = 30; follow-up is 7–10 years after the baseline).

	Baseline	Follow-up
Male nr. (%)	23 (77%)	n/a
Age (years)	57 ± 9	65 ± 9
Verhage education score (1/2/3/4/5/6/7)	0/1/0/4/11/3/11	n/a
Disease duration (years)	5 ± 5	14 ± 5
H and Y stage (1/1.5/2/2.5/3/4/5)	4/5/16/4/1/0/0	0/0/13/7/4/6/0 **
UPDRS-III score	20 ± 10	26 ± 14 *
MMSE score	29 ± 2	29 ± 2
LEDD	548 ± 481	1075 ± 455
Cognitive status (normal/MCI/PDD)	29/0/1	16/5/9 **

Data are presented as mean ± standard deviation unless indicated otherwise. Abbreviations: H and Y stage = Hoehn and Yahr stage, UPDRS-III = Unified Parkinson’s Disease Rating Scale III, MMSE = Mini-Mental State Examination, LEDD = Levodopa Equivalent Daily Dose, MCI = Mild Cognitive Impairment, PDD = Parkinson’s disease dementia, n/a = not applicable. * Difference is statistically significant (*p* < 0.05). ** Difference is statistically significant (*p* < 0.001).

## Data Availability

The datasets generated and analyzed during the current study are available from the corresponding author upon reasonable request. Proteomic validation data used in the preparation of this article were obtained from the Accelerating Medicine Partnership^®^ (AMP^®^) Parkinson’s Disease (AMP PD) Knowledge Platform. For up-to-date information on the study, visit https://www.amp-pd.org accessed on 1 July 2023. The AMP^®^ PD program is a public–private partnership managed by the Foundation for the National Institutes of Health and funded by the National Institute of Neurological Disorders and Stroke (NINDS) in partnership with the Aligning Science Across Parkinson’s (ASAP) initiative; Celgene Corporation, a subsidiary of Bristol-Myers Squibb Company; GlaxoSmithKline plc (GSK); The Michael J. Fox Foundation for Parkinson’s Research; Pfizer Inc.; Sanofi US Services Inc.; and Verily Life Sciences. ACCELERATING MEDICINES PARTNERSHIP and AMP are registered service marks of the U.S. Department of Health and Human Services.

## Supplementary Materials

The following supporting information can be downloaded at: https://www.mdpi.com/article/10.3390/ijms241914915/s1.

## References

[1] Tan E.K., Chao Y.X., West A., Chan L.L., Poewe W., Jankovic J. (2020). Parkinson disease and the immune system—Associations, mechanisms and therapeutics. Nat. Rev. Neurol..

[2] Bartl M., Xylaki M., Bähr M., Weber S., Trenkwalder C., Mollenhauer B. (2022). Evidence for immune system alterations in peripheral biological fluids in Parkinson’s disease. Neurobiol. Dis..

[3] McGeer P.L., Itagaki S., Boyes B.E., McGeer E.G. (1988). Reactive microglia are positive for HLA-DR in the substantia nigra of Parkinson’s and Alzheimer’s disease brains. Neurology.

[4] Galiano-Landeira J., Torra A., Vila M., Bove J. (2020). CD8 T cell nigral infiltration precedes synucleinopathy in early stages of Parkinson’s disease. Brain.

[5] Sulzer D., Alcalay R.N., Garretti F., Cote L., Kanter E., Agin-Liebes J., Liong C., McMurtrey C., Hildebrand W.H., Mao X. (2017). T cells from patients with Parkinson’s disease recognize alpha-synuclein peptides. Nature.

[6] Reale M., Iarlori C., Thomas A., Gambi D., Perfetti B., Di Nicola M., Onofrj M. (2009). Peripheral cytokines profile in Parkinson’s disease. Brain Behav. Immun..

[7] Brodacki B., Staszewski J., Toczylowska B., Kozlowska E., Drela N., Chalimoniuk M., Stepien A. (2008). Serum interleukin (IL-2, IL-10, IL-6, IL-4), TNFalpha, and INFgamma concentrations are elevated in patients with atypical and idiopathic parkinsonism. Neurosci. Lett..

[8] Rathnayake D., Chang T., Udagama P. (2019). Selected serum cytokines and nitric oxide as potential multi-marker biosignature panels for Parkinson disease of varying durations: A case-control study. BMC Neurol..

[9] Liu T.W., Chen C.M., Chang K.H. (2022). Biomarker of Neuroinflammation in Parkinson’s Disease. Int. J. Mol. Sci..

[10] Usenko T.S., Nikolaev M.A., Miliukhina I.V., Bezrukova A.I., Senkevich K.A., Gomzyakova N.A., Beltceva Y.A., Zalutskaya N.M., Gracheva E.V., Timofeeva A.A. (2020). Plasma cytokine profile in synucleinophaties with dementia. J. Clin. Neurosci..

[11] Brockmann K., Schulte C., Schneiderhan-Marra N., Apel A., Pont-Sunyer C., Vilas D., Ruiz-Martinez J., Langkamp M., Corvol J.C., Cormier F. (2017). Inflammatory profile discriminates clinical subtypes in LRRK2-associated Parkinson’s disease. Eur. J. Neurol..

[12] Williams-Gray C.H., Wijeyekoon R., Yarnall A.J., Lawson R.A., Breen D.P., Evans J.R., Cummins G.A., Duncan G.W., Khoo T.K., Burn D.J. (2016). Serum immune markers and disease progression in an incident Parkinson’s disease cohort (ICICLE-PD). Mov. Disord..

[13] Bartl M., Dakna M., Schade S., Otte B., Wicke T., Lang E., Starke M., Ebentheuer J., Weber S., Toischer K. (2023). Blood Markers of Inflammation, Neurodegeneration, and Cardiovascular Risk in Early Parkinson’s Disease. Mov. Disord..

[14] Abdi I.Y., Bartl M., Dakna M., Abdesselem H., Majbour N., Trenkwalder C., El-Agnaf O., Mollenhauer B. (2023). Cross-sectional proteomic expression in Parkinson’s disease-related proteins in drug-naive patients vs healthy controls with longitudinal clinical follow-up. Neurobiol. Dis..

[15] Winchester L., Barber I., Lawton M., Ash J., Liu B., Evetts S., Hopkins-Jones L., Lewis S., Bresner C., Malpartida A.B. (2023). Identification of a possible proteomic biomarker in Parkinson’s disease: Discovery and replication in blood, brain and cerebrospinal fluid. Brain Commun..

[16] Parkinson Progression Marker I. (2011). The Parkinson Progression Marker Initiative (PPMI). Prog. Neurobiol..

[17] Neal M.L., Fleming S.M., Budge K.M., Boyle A.M., Kim C., Alam G., Beier E.E., Wu L.J., Richardson J.R. (2020). Pharmacological inhibition of CSF1R by GW2580 reduces microglial proliferation and is protective against neuroinflammation and dopaminergic neurodegeneration. FASEB J..

[18] Chandrasekaran S., Bonchev D. (2013). A network view on Parkinson’s disease. Comput. Struct. Biotechnol. J..

[19] Yun H.M., Kim J.A., Hwang C.J., Jin P., Baek M.K., Lee J.M., Hong J.E., Lee S.M., Han S.B., Oh K.W. (2015). Neuroinflammatory and Amyloidogenic Activities of IL-32beta in Alzheimer’s Disease. Mol. Neurobiol..

[20] Alrafiah A., Al-Ofi E., Obaid M.T., Alsomali N. (2019). Assessment of the Levels of Level of Biomarkers of Bone Matrix Glycoproteins and Inflammatory Cytokines from Saudi Parkinson Patients. Biomed. Res. Int..

[21] Koper O.M., Kaminska J., Sawicki K., Kemona H. (2018). CXCL9, CXCL10, CXCL11, and their receptor (CXCR3) in neuroinflammation and neurodegeneration. Adv. Clin. Exp. Med..

[22] Lin Y., Zhou M., Dai W., Guo W., Qiu J., Zhang Z., Mo M., Ding L., Ye P., Wu Y. (2021). Bone-Derived Factors as Potential Biomarkers for Parkinson’s Disease. Front. Aging Neurosci..

[23] Lindestam Arlehamn C.S., Dhanwani R., Pham J., Kuan R., Frazier A., Rezende Dutra J., Phillips E., Mallal S., Roederer M., Marder K.S. (2020). alpha-Synuclein-specific T cell reactivity is associated with preclinical and early Parkinson’s disease. Nat. Commun..

[24] Stevens C.H., Rowe D., Morel-Kopp M.C., Orr C., Russell T., Ranola M., Ward C., Halliday G.M. (2012). Reduced T helper and B lymphocytes in Parkinson’s disease. J. Neuroimmunol..

[25] Cleary A.M., Tu W., Enright A., Giffon T., Dewaal-Malefyt R., Gutierrez K., Lewis D.B. (2003). Impaired accumulation and function of memory CD4 T cells in human IL-12 receptor beta 1 deficiency. J. Immunol..

[26] Walsh M.C., Choi Y. (2021). Regulation of T cell-associated tissues and T cell activation by RANKL-RANK-OPG. J. Bone Miner. Metab..

[27] Muller M., Carter S., Hofer M.J., Campbell I.L. (2010). Review: The chemokine receptor CXCR3 and its ligands CXCL9, CXCL10 and CXCL11 in neuroimmunity—A tale of conflict and conundrum. Neuropathol. Appl. Neurobiol..

[28] Reynolds A.D., Banerjee R., Liu J., Gendelman H.E., Mosley R.L. (2007). Neuroprotective activities of CD4+CD25+ regulatory T cells in an animal model of Parkinson’s disease. J. Leukoc. Biol..

[29] Simats A., Garcia-Berrocoso T., Penalba A., Giralt D., Llovera G., Jiang Y., Ramiro L., Bustamante A., Martinez-Saez E., Canals F. (2018). CCL23: A new CC chemokine involved in human brain damage. J. Intern. Med..

[30] Faura J., Bustamante A., Penalba A., Giralt D., Simats A., Martinez-Saez E., Alcolea D., Fortea J., Lleo A., Teunissen C.E. (2020). CCL23: A Chemokine Associated with Progression from Mild Cognitive Impairment to Alzheimer’s Disease. J. Alzheimer’s Dis..

[31] Umeh C.C., Mahajan A., Mihailovic A., Pontone G.M. (2022). APOE4 Allele, Sex, and Dementia Risk in Parkinson’s Disease: Lessons From a Longitudinal Cohort. J. Geriatr. Psychiatry Neurol..

[32] Yu J.T., Tan L., Hardy J. (2014). Apolipoprotein E in Alzheimer’s disease: An update. Annu. Rev. Neurosci..

[33] Yeo Y.A., Martinez Gomez J.M., Croxford J.L., Gasser S., Ling E.A., Schwarz H. (2012). CD137 ligand activated microglia induces oligodendrocyte apoptosis via reactive oxygen species. J. Neuroinflamm..

[34] Wong H.Y., Prasad A., Gan S.U., Chua J.J.E., Schwarz H. (2020). Identification of CD137-Expressing B Cells in Multiple Sclerosis Which Secrete IL-6 Upon Engagement by CD137 Ligand. Front. Immunol..

[35] Guler S., Gul T., Guler S., Haerle M.C., Basak A.N. (2021). Early-Onset Parkinson’s Disease: A Novel Deletion Comprising the DJ-1 and TNFRSF9 Genes. Mov. Disord..

[36] Ma Y.J., Berg-von der Emde K., Moholt-Siebert M., Hill D.F., Ojeda S.R. (1994). Region-specific regulation of transforming growth factor alpha (TGF alpha) gene expression in astrocytes of the neuroendocrine brain. J. Neurosci..

[37] Mogi M., Harada M., Narabayashi H., Inagaki H., Minami M., Nagatsu T. (1996). Interleukin (IL)-1 beta, IL-2, IL-4, IL-6 and transforming growth factor-alpha levels are elevated in ventricular cerebrospinal fluid in juvenile parkinsonism and Parkinson’s disease. Neurosci. Lett..

[38] Gigase F.A.J., Smith E., Collins B., Moore K., Snijders G., Katz D., Bergink V., Perez-Rodriquez M.M., De Witte L.D. (2023). The association between inflammatory markers in blood and cerebrospinal fluid: A systematic review and meta-analysis. Mol. Psychiatry.

[39] Lan G., Wang P., Chan R.B., Liu Z., Yu Z., Liu X., Yang Y., Zhang J. (2022). Astrocytic VEGFA: An essential mediator in blood-brain-barrier disruption in Parkinson’s disease. Glia.

[40] Wada K., Arai H., Takanashi M., Fukae J., Oizumi H., Yasuda T., Mizuno Y., Mochizuki H. (2006). Expression levels of vascular endothelial growth factor and its receptors in Parkinson’s disease. Neuroreport.

[41] Trares K., Bhardwaj M., Perna L., Stocker H., Petrera A., Hauck S.M., Beyreuther K., Brenner H., Schottker B. (2022). Association of the inflammation-related proteome with dementia development at older age: Results from a large, prospective, population-based cohort study. Alzheimer’s Res. Ther..

[42] Larsson A., Carlsson L., Gordh T., Lind A.L., Thulin M., Kamali-Moghaddam M. (2015). The effects of age and gender on plasma levels of 63 cytokines. J. Immunol. Methods.

[43] Cerri S., Mus L., Blandini F. (2019). Parkinson’s Disease in Women and Men: What’s the Difference?. J. Park. Dis..

[44] Espay A.J., Schwarzschild M.A., Tanner C.M., Fernandez H.H., Simon D.K., Leverenz J.B., Merola A., Chen-Plotkin A., Brundin P., Kauffman M.A. (2017). Biomarker-driven phenotyping in Parkinson’s disease: A translational missing link in disease-modifying clinical trials. Mov. Disord..

[45] van Dijk K.D., Jongbloed W., Heijst J.A., Teunissen C.E., Groenewegen H.J., Berendse H.W., van de Berg W.D., Veerhuis R. (2013). Cerebrospinal fluid and plasma clusterin levels in Parkinson’s disease. Park. Relat. Disord..

[46] Hughes A.J., Daniel S.E., Kilford L., Lees A.J. (1992). Accuracy of clinical diagnosis of idiopathic Parkinson’s disease: A clinico-pathological study of 100 cases. J. Neurol. Neurosurg. Psychiatry.

[47] Dalrymple-Alford J.C., Livingston L., MacAskill M.R., Graham C., Melzer T.R., Porter R.J., Watts R., Anderson T.J. (2011). Characterizing mild cognitive impairment in Parkinson’s disease. Mov. Disord..

[48] Litvan I., Goldman J.G., Troster A.I., Schmand B.A., Weintraub D., Petersen R.C., Mollenhauer B., Adler C.H., Marder K., Williams-Gray C.H. (2012). Diagnostic criteria for mild cognitive impairment in Parkinson’s disease: Movement Disorder Society Task Force guidelines. Mov. Disord..

[49] Olde Dubbelink K.T., Stoffers D., Deijen J.B., Twisk J.W., Stam C.J., Berendse H.W. (2013). Cognitive decline in Parkinson’s disease is associated with slowing of resting-state brain activity: A longitudinal study. Neurobiol. Aging.

[50] Wik L., Nordberg N., Broberg J., Bjorkesten J., Assarsson E., Henriksson S., Grundberg I., Pettersson E., Westerberg C., Liljeroth E. (2021). Proximity Extension Assay in Combination with Next-Generation Sequencing for High-throughput Proteome-wide Analysis. Mol. Cell Proteom..

[51] Aarsland D., Creese B., Politis M., Chaudhuri K.R., Ffytche D.H., Weintraub D., Ballard C. (2017). Cognitive decline in Parkinson disease. Nat. Rev. Neurol..

[52] Blauwendraat C., Faghri F., Pihlstrom L., Geiger J.T., Elbaz A., Lesage S., Corvol J.C., May P., Nicolas A., Abramzon Y. (2017). NeuroChip, an updated version of the NeuroX genotyping platform to rapidly screen for variants associated with neurological diseases. Neurobiol. Aging.

[53] Johnston R., Jones K., Manley D. (2018). Confounding and collinearity in regression analysis: A cautionary tale and an alternative procedure, illustrated by studies of British voting behaviour. Qual. Quant..

