# Supplementary material for: Inflammatory Blood Biomarkers Are Associated with Long-Term Clinical Disease Severity in Parkinson’s Disease
Source: Int J Mol Sci. 2023 Oct 5;24(19):14915. doi: 10.3390/ijms241914915 (PMC10573398; doi:10.3390/ijms241914915)
Supplement: Supplementary file 1 [file ijms-24-14915-s001.zip › ijms-2539460-supplementary.pdf]

Supplementary File S1

For validation purposes we used Olink targeted proteomics data of the Accelerating Medicines Partnership Parkinson's Disease (AMP-PD) program, which consists of data from the Parkinson's Disease Biomarkers Program (PDBP) cohort and the Parkinson's Progression Markers Initiative (PPMI) cohort. The PDBP cohort is comprised of PD participants recruited at 10 academic centers in the United States across 11 research projects. All projects are observational studies that share 2 inclusion criteria for the PD cohorts: diagnosis of PD based on UK PD Society Brain Bank criteria [1], and the ability to provide written informed consent or have an informed consent proxy. For more information on the PDBP study, please visit this link (https://amp-pd.org/unified-cohorts/pdbp#study-overview). The Parkinson's Progression Marker Initiative (PPMI) is a prospective, longitudinal, observational, international multicenter study. All PD participants have a clinical diagnosis of PD and a positive dopamine transporter (DAT) SPECT scan. Details of the study and the entire protocol can be viewed here: www.ppmi-info.org/ study-design and have been published previously [2]. For this manuscript, data from version 2.5 of the AMP-PD cohort was used.

For the current analysis, all subjects who had PD listed as their primary diagnosis, and who had completed a baseline and 48 months follow-up study visit at the time of AMP-PD data download (November 15th, 2022) were included (Supplementary Table 1). To evaluate differences between PD and controls, we performed an ANOVA with age as confounder. The following proteins were altered in PD compared to controls in the independent validation cohort; CCL20, CCL3, FGF5, IL17A, MMP10, TNF and TNFSF10. However, the differences were not significant after multiple comparison correction.

For the longitudinal analysis, a paired t-test was used to calculate differences between PD patients and controls over time. Motor symptoms (UPDRS-III and H&Y stage; p=0.01 and p<0.001, respectively) were significantly more severe at follow-up measurements after 48 months, compared to baseline. The level of 24 proteins significantly changed over time, of which 2 were also increased in our analysis, i.e. CCL25 (t=-2.13, df 125.89, p= 0.035) and TGF-alpha (t=-2.47, df=128.34, p=0.014). The increase in level of VEGFA that we found reached only trend level in the validation cohort (t=-1.93, df 139.28,

p= 0.056; supplementary Figure 1). No correlation was found between the increased level of TGF-alpha and higher UPDRS III scores at follow-up in the validation cohort (only available of N=56).

Table S1. Demographics of the AMP-PD validation cohort at baseline

	**PD patients**	**Healthy controls**
N	105	83
**Male** nr. (%)	74 (70%)	51 (61%)
**Age** (years)	64 ± 10	62 ± 11
**Disease duration** (years; N=67)	5 ± 5	n/a
**H&Y** stage (1/2/3/4/5) baseline (N=105)	19/76/8/2/0	n/a
**H&Y** stage (1/2/3/4/5) follow-up 48 months (N=72)	3/61/4/3/1**	n/a
**UPDRS-III** score baseline (N=105)	25 ± 11	n/a
**UPDRS-III** score follow-up 48 months (N=72)	29 ± 13*	n/a

Legend: data is presented as mean ± standard deviation unless indicated otherwise. Abbreviations: H&Y stage = Hoehn and Yahr stage, UPDRS-III = Unified Parkinson's Disease Rating Scale III, n/a = not applicable.

* Difference is statistically significant compared to baseline ($p < 0.05$)

** Difference is statistically significant compared to baseline ($p < 0.001$)

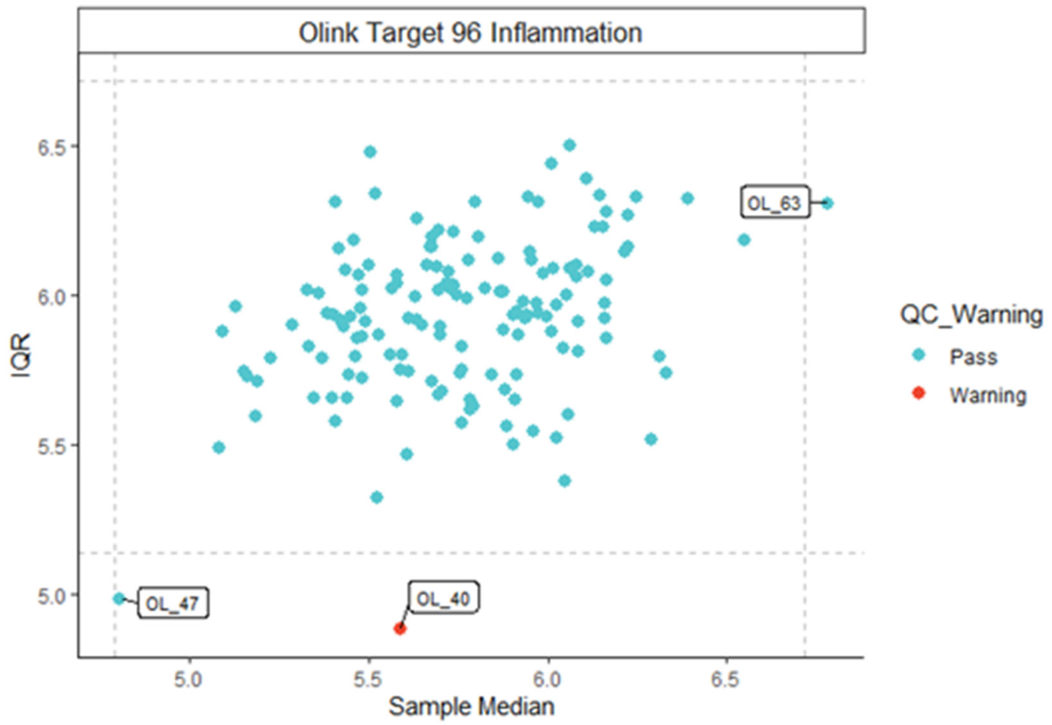

Figure S1. *Legend:* Quality control plot showing the results of the cross-sectional analysis. Horizontal dashed lines indicate +/- 3 standard deviations from the mean interquartile range. Vertical dashed lines indicate +/- 3 standard deviations from the mean sample median. One unsuccessful measurement (labelled red dot) and two outliers (labelled blue dots) were identified. These samples were excluded from the statistical analysis.

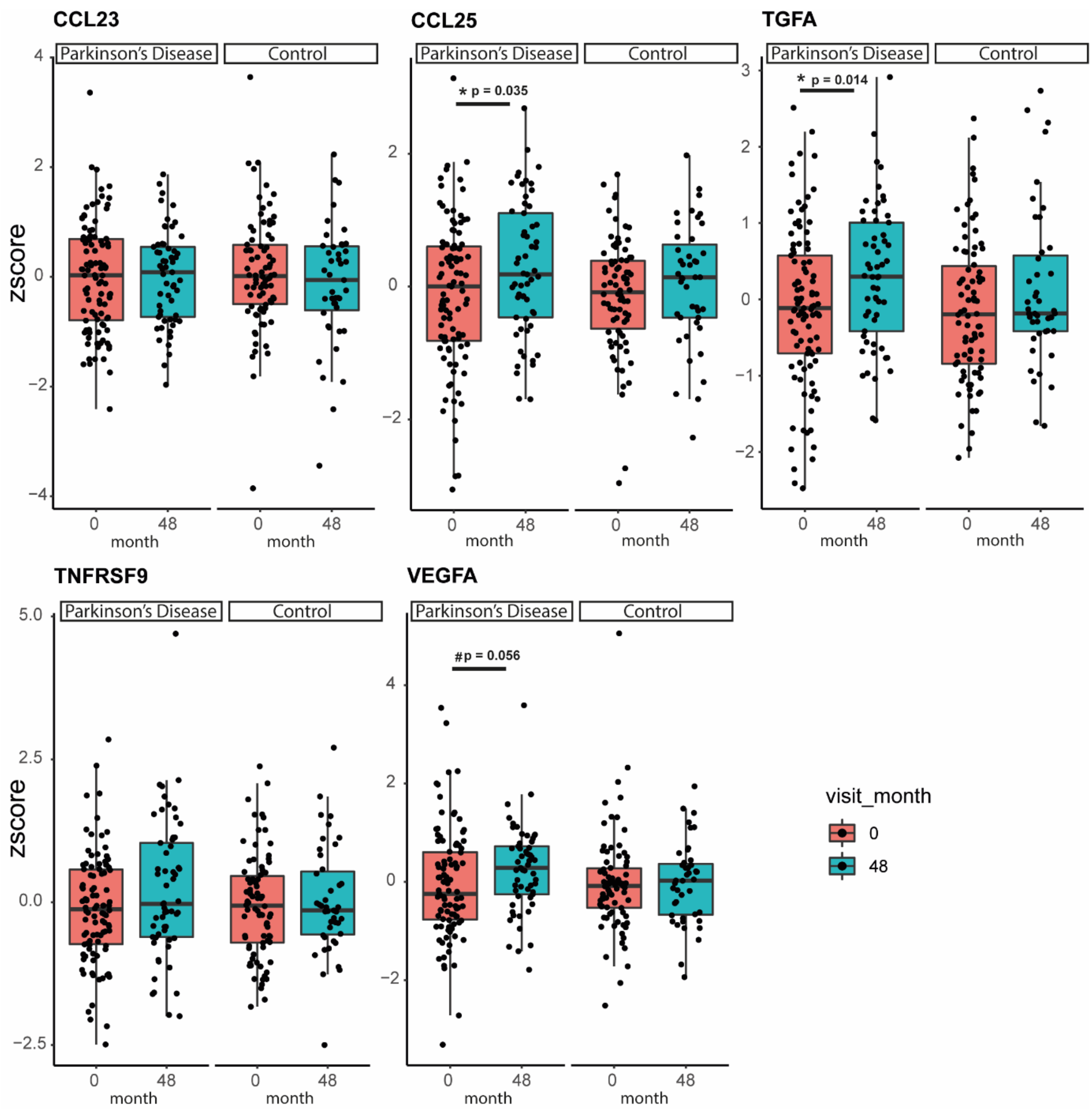

Figure S2. *Legend:* Longitudinal data (follow-up time: 48 months) of the 5 proteins in the validation cohort, that were significantly higher after follow-up in our cohort. The level of 2 proteins significantly increased over time, i.e. CCL25 and TGF-alpha; the increase in level of VEGFA reached only trend level. Of these 5 proteins, only CCL25 significantly increased as well in control subjects.

1. Hughes, A.J., et al., *Accuracy of clinical diagnosis of idiopathic Parkinson's disease: a clinico-pathological study of 100 cases.* J. Neurol. Neurosurg. Psychiatry, 1992. **55**(3): p. 181-184.
2. Parkinson Progression Marker, I., *The Parkinson Progression Marker Initiative (PPMI).* Prog Neurobiol, 2011. **95**(4): p. 629-35.
